# Extrafacial Acquired Dermal Melanocytosis: An Exceptionally Rare Example With Bilateral Multifocal Disease

**DOI:** 10.7759/cureus.61284

**Published:** 2024-05-29

**Authors:** Umar A Hussain, Kruti Gandhi, Jamie A Tschen

**Affiliations:** 1 Histopathology, Manchester University National Health Service (NHS) Foundation Trust, Manchester, GBR; 2 Dermatology, DermSurgery Associates-Pearland, Houston, USA; 3 Dermatopathology, St. Joseph Hospital, Houston, USA

**Keywords:** acquired, extrafacial, elastolysis, multifocal involvement, naevus of ota, hori naevus, mongolian spot, dermal melanocytosis

## Abstract

Dermal melanocytoses are a group of cutaneous disorders characterized by the presence of ectopic melanocytes in the dermis; the most well-known example is the Mongolian spot. Acquired dermal melanocytosis (ADM) is a term used to describe the onset of dermal melanocytosis occurring after its usual age of presentation (i.e., birth and infancy). ADMs usually occur on the face and can less commonly affect extrafacial sites, such as the back and limbs. Purely extrafacial ADM is extremely uncommon and, when present, is usually unifocal. Herein, we present an exceptionally rare example of purely extrafacial ADM with extensive bilateral involvement in a 44-year-old female originally from the Philippines.

## Introduction

Dermal melanocytoses are a group of cutaneous disorders defined by the presence of melanocytes in the dermis [1]. They are generally considered a type of birthmark, as most will present at birth or during infancy as flat, ill-defined, bluish-grey skin lesions [1]. Acquired dermal melanocytosis (ADM) describes the presence of dermal melanocytosis outside of the typical age presentation. When this occurs, the face is frequently involved [2]. Purely extrafacial ADMs are rare and, when present, are usually unifocal [3]. Herein, we present an exceptionally rare example of purely extrafacial ADM presenting with extensive bilateral disease in a 44-year-old female.

## Case presentation

**Figure 1:**
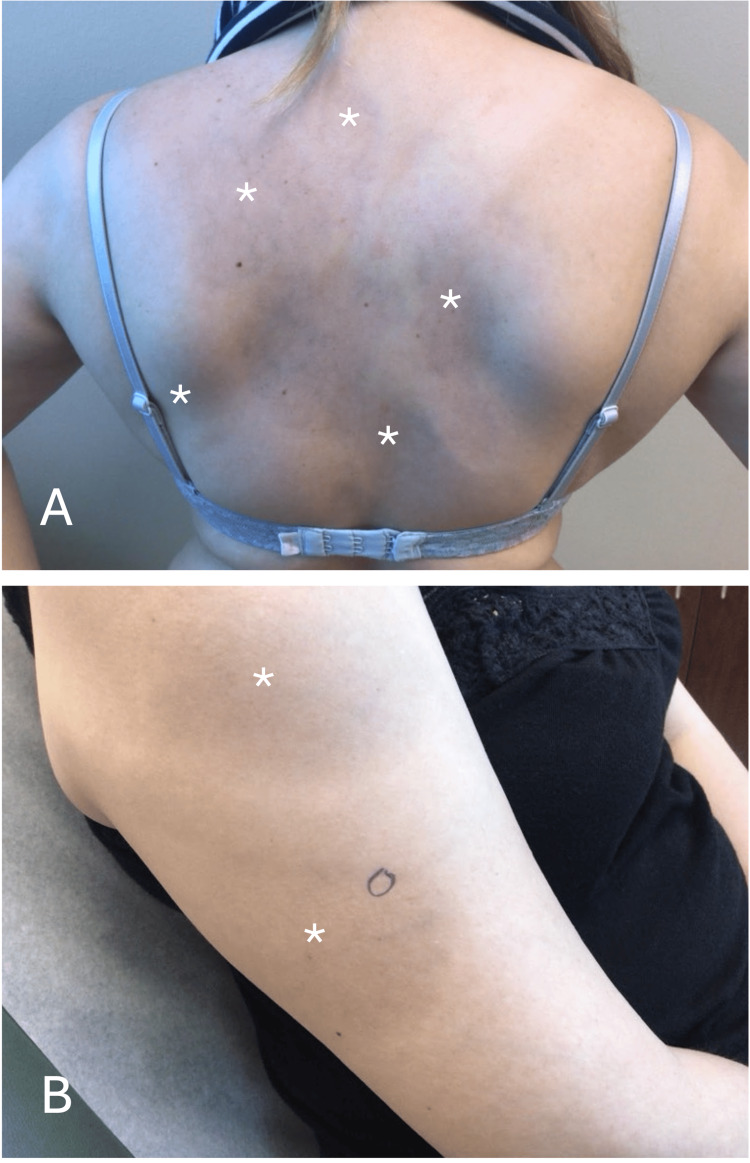
Multiple non-communicating irregular bluish-grey lesions are seen on both sides of the patient’s back (A), right arm, and right shoulder (B). Each lesion has been marked (*) at its approximate epicenter for convenience. Similar lesions were present on the opposite arm and both legs. A punch biopsy was taken from the area within the inked oval on the right arm (B).

A 44-year-old female with a 30-plus-year history of skin pigmentation came to our dermatology clinic for evaluation. She was born in the Philippines and moved to Texas in 2005. She had a previous biopsy in the Philippines at 18 years old from the initial lesion on the back, which was reported as “skin pigmentation." She requested further investigation as she had noticed similar hyperpigmented patches developing on her arms, back, and legs since then (Figure 1). On examination, there were approximately 10 hyperpigmented patches on the bilateral arms, back, and bilateral legs, all of which varied in size. The lesions were not itchy or painful, and according to the patient, they had been present for over 30 years. There was no family history of melanocytosis. Past medical history included Hashimoto thyroiditis, hypercholesterolemia, hypertension, and allergic rhinitis. Medication history included rosuvastatin, telmisartan, and montelukast. Clinical differential diagnosis included atrophoderma of Pasini and Pierini, morphoea, erythema dyschromicum perstans (ashy dermatosis), and dermal melanocytosis. 

**Figure 2:**
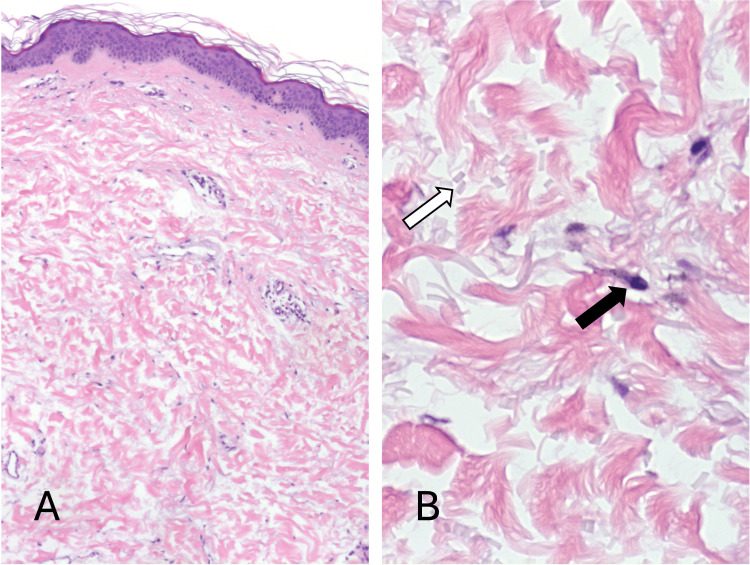
A) A low-power view of the punch biopsy shows relatively unremarkable skin. B) Higher magnification of the reticular dermis reveals pigmented spindled cells (black arrow) with fragmented elastic fibers (white arrow) in close approximation

**Figure 3:**
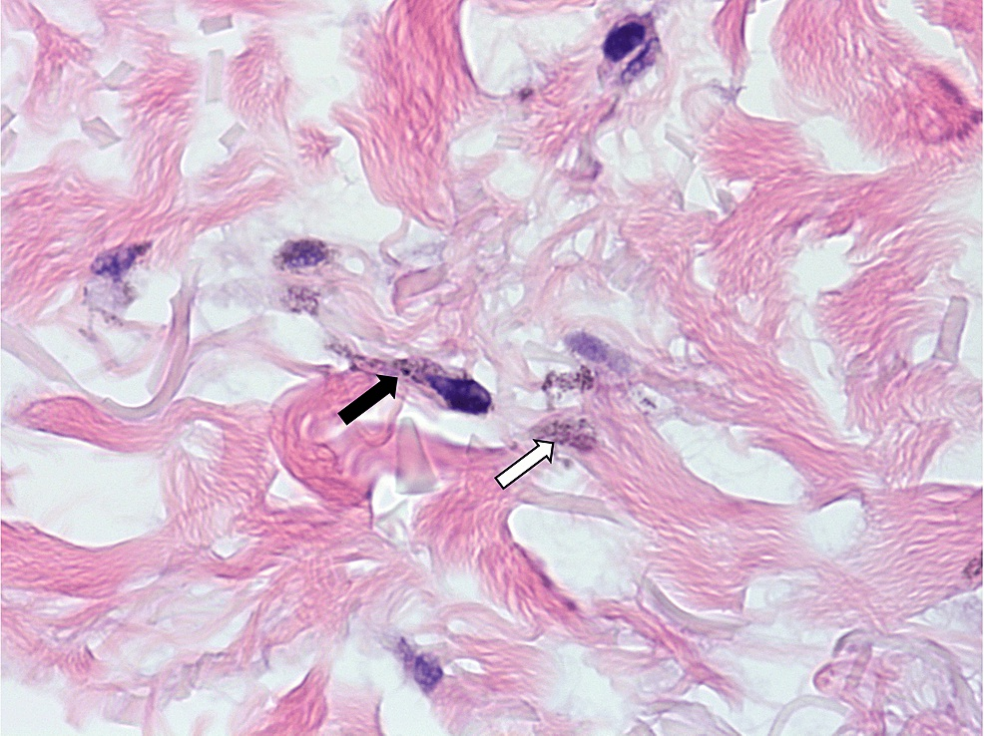
A high-power view demonstrates fine melanin pigment present in the cytoplasm of the melanocyte (black arrow). Some pigment is also appreciated extracellularly amongst collagen bundles (white arrow). Fragmented elastic fibers are present in the top-left of the figure.

**Figure 4:**
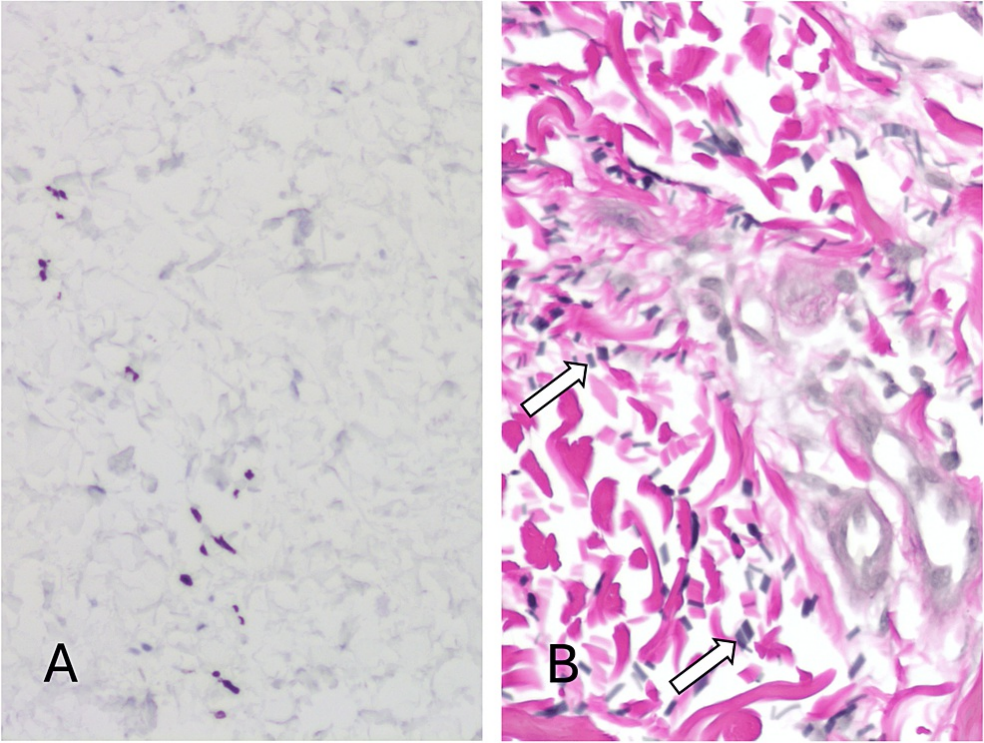
A) The immunohistochemical stain MART-1 (Melan-A) highlights numerous spindle cells in the dermis (brown), confirming their melanocytic nature. B) Verhoeff Van Gieson stain demonstrates numerous black-staining elastin fiber fragments (white arrows) consistent with elastolysis. The background collagen stains pink.

A punch biopsy of the skin was taken from a representative area of the lesion and sent to pathology. Histology showed a normal-thickness epidermis with orthokeratosis. At low power, the dermis appeared relatively unremarkable, with normal-appearing collagen bundles (Figure 2A). There was a very mild superficial perivascular infiltrate composed of mononuclear cells. Higher-power magnification revealed scattered pigment-bearing spindle cells occupying the mid-dermis, which possessed hyperchromatic but relatively uniform nuclei (Figures 2B, 3). No mitotic figures were identified. The spindle cells were highlighted with MART-1 (Melan-A), confirming their melanocytic nature (Figure 4A). The histological features were consistent with dermal melanocytosis. In addition, the background superficial dermis exhibited conspicuous elastic fiber fragmentation in keeping with elastolysis, which was confirmed with Verhoeff-Van Gieson stain (Figures 2B, 4B). The patient was counseled and offered laser therapy, which she is currently considering. 

## Discussion

Dermal melanocytoses encompass a group of cutaneous disorders characterized by ectopic dendritic melanocytes situated in the dermis [1]. The melanocytes preferentially refract shorter-wavelength light rays (i.e., blue and violet) across a field of skin, producing the characteristic blue-grey hue associated with these skin lesions [4]. The pathogenesis is believed to be related to incomplete neural crest cell migration during fetal development, but other theories, including melanocytes “dropping off” from the epidermal and follicular epithelium, have also been proposed [4,5]. 

Dermal melanocytoses are subclassified based on their clinical distribution (Table 1). They can occur in any race but are particularly common in patients of Asian, African, or Hispanic descent [1,6]. Rarely reported associations include diffuse systemic sclerosis, lysosomal storage diseases (such as Hurler syndrome and GM1 gangliosidosis), and malignant transformation to melanoma [4,5,7-9]. In most cases, however, no such association is present, and the concern is largely cosmetic, for which repeated bouts of laser therapy are considered the most effective treatment option [10].

**Table 1:** Subtypes of dermal melanocytoses and their typical distributions

Subtype of dermal melanocytosis	Typical distribution [1,4]
Mongolian Spot	Mostly lumbosacral and buttocks; can occur anywhere
Nevus of Ota	Unilateral along trigeminal distribution (typically V_1_ and V_2_)
Nevus of Hori (Sun Nevus)	Bilateral along trigeminal distribution (typically V_1_ and V_2_)
Nevus of Ito	Unilateral neck/supraclavicular/scapula (acromioclavicular)
Dermal melanocyte hamartoma	Follows dermatomal distribution
Blue nevus	Presents as a tumour on the buttocks, head and neck and distal extremities

Dermal melanocytoses typically appear at birth or in the first few weeks of life. When they present outside of this time, they are generally considered to be acquired. Acquired dermal melanocytosis (ADM) is a rare phenomenon and typically involves the face (i.e., Nevus of Ota and Nevus of Hori) and may, in addition, affect extrafacial sites [2,3]. Purely extrafacial ADM is particularly rare and, when present, is almost always unifocal [3]. A review of literature revealed only one other case of purely extrafacial ADM with multifocal and bilateral distribution - that was a case involving the dorsum of both feet in a 69-year-old female [3]. Our patient, in contrast, had a particularly extensive case of extrafacial ADM, the likes of which have not previously been reported in the literature. Consideration was given to the possibility of connective tissue pathologies, such as atrophoderma or scleroderma, given the atypicality of the presentation, but the histological features did not support this.

A supplementary feature in this case was the presence of superficial dermal elastolysis, a morphological phenomenon describing the fragmentation of dermal elastic fibers. It is associated with multiple elastic tissue disorders and usually corresponds to lax or wrinkly skin, but in this case, it was subclinical. Given that the lesion was present at a sun-exposed site and the lack of significant associated clinical findings, we feel this was most likely an incidental finding associated with sun damage. 

## Conclusions

Extrafacial ADM is a rare presentation of dermal melanocytosis that typically presents in an unifocal manner. Bilateral, extensive involvement, as observed in this case, is extremely rare. Histological examination was instrumental in making the diagnosis, given the atypicality of the presentation.

## References

[1] Franceschini D, Dinulos JG (2015). Dermal melanocytosis and associated disorders. Curr Opin Pediatr.

[2] Murakami F, Soma Y, Mizoguchi M (2005). Acquired symmetrical dermal melanocytosis (naevus of Hori) developing after aggravated atopic dermatitis. Br J Dermatol.

[3] Lee JY, Lee JS, Kim YC (2010). Histopathological features of acquired dermal melanocytosis. Eur J Dermatol.

[4] Stanford DG, Georgouras KE (1996). Dermal melanocytosis: a clinical spectrum. Australas J Dermatol.

[5] Hori Y, Kawashima M, Oohara K, Kukita A (1984). Acquired, bilateral nevus of Ota-like macules. J Am Acad Dermatol.

[6] Jacobs AH, Walton RG (1976). The incidence of birthmarks in the neonate. Pediatrics.

[7] Bisceglia M, Carosi I, Fania M, Di Ciommo A, Lomuto M (1997). [Nevus of Ota. Presentation of a case associated with a cellular blue nevus with suspected malignant degeneration and review of the literature]. Pathologica.

[8] Martínez-Peñuela A, Iglesias ME, Mercado MR, Martínez-Peñuela JM (2011). [Malignant transformation of a nevus of Ito: description of a rare case]. Actas Dermosifiliogr.

[9] Hanson M, Lupski JR, Hicks J, Metry D (2003). Association of dermal melanocytosis with lysosomal storage disease: clinical features and hypotheses regarding pathogenesis. Arch Dermatol.

[10] Yoshimura K, Sato K, Aiba-Kojima E (2006). Repeated treatment protocols for melasma and acquired dermal melanocytosis. Dermatol Surg.

